# Retrospective Case-Control Study of Apolipoprotein J/Clusterin Protein Expression in Early Liveborn Neonatal Deaths with and without Pontosubicular Necrosis

**DOI:** 10.1155/2012/479359

**Published:** 2012-07-12

**Authors:** Kathreena M. Kurian, Declan McGuone

**Affiliations:** ^1^Department of Neuropathology, Frenchay Hospital, Frenchay Park Road, Frenchay, Bristol BS16 1LE, UK; ^2^Department of Neuropathology, Massachusetts General Hospital, 55 Fruit Street, Boston, MA 02114, USA

## Abstract

*Aims*. Our objective was to examine Apo J protein expression in a total of 27 early liveborn neonatal deaths (less than 7 days of age) selected from the Scottish Perinatal Study (gestation of 25–42 weeks) comparing a group with histological pontosubicular necrosis (PSN) (*n* = 12) to a control group lacking PSN (*n* = 15). *Methods*. Using immunohistochemistry we evaluated postmortem pons and hippocampus from patients with PSN versus controls. *Results*. In the group with PSN, 11/12 (92%) cases showed positive Apo J neurones in the hippocampus/pons compared with 6/15 (40%) cases without PSN (*P* = 0.014, odds ratio 27.5, 95% confidence interval 2.881–262.48, using exact logistic regression)—independent of gestation, presence or absence of clinical asphyxia, duration of labour, or postnatal age. Clinical asphyxia was present in 10/15 (67%) without PSN compared with 11/12 (92%) with PSN. Neuronal Apo J positivity was present in 15/21 (71%) of clinically asphyxiated cases compared with 2/6 (33%) of the cases with no evidence of clinical asphyxia (*P* = 0.154, odds ratio 5, 95% confidence interval 0.71 to 34.94). *Conclusions*. Apo J neuronal protein expression is significantly increased in cases with PSN compared to cases without PSN—independent of gestation, presence of clinical asphyxia, duration of labour, or postnatal age.

## 1. Introduction

 Apolipoprotein J (Apo J), (also known as clusterin, serum protein 40, cytolysis inhibitor CLI, glycoprotein III, sulfated glycoprotein 2 SGP 2, or testosterone repressed prostate message 2, TRPM-2) was first identified from chromaffin granules of the bovine adrenal medulla and in the ram rete testis [1, 2]. Like Apolipoprotein E, it is expressed in the brain in a number of physiological and pathological conditions [3]. Apo J has been implicated in cell-cell interactions, lipid transport, inhibition of complement-mediated cell lysis, secretion, hypoxic/ischaemic encephalopathy, epilepsy, apoptosis, and neurodegeneration [4–12]. A truncated intracellular form that lacks the signal peptide for processing in the endoplasmic reticulum has also been described [13].

At a molecular level, Han et al. have shown in an experimental animal model of HI that Apo J accumulates in dying neurones and that Apo J-null mice suffered 50% less brain injury compared with wild-type mice subjected to HI (hypoxic/ischaemic) insults [14].

Pontosubicular necrosis (PSN) describes the association on histology between hypoxic death of neurones in the ventral pons and the subiculum in human neonates. Experimental studies have shown that the mode of death in these neurons is apoptosis [15]. We examined Apo J protein expression in the pons and hippocampus of early liveborn neonatal deaths comparing 12 cases with PSN to 15 controls lacking PSN on histology. Our null hypothesis predicted no significant difference in Apo J protein expression between both groups. To our knowledge the expression of Apo J has not been examined previously in early neonatal deaths.

## 2. Methods

27 cases were selected from the Scottish Perinatal Neuropathology Study, which included Scottish early liveborn neonatal deaths (less than 7 days of age) with the exclusion of infants with known chromosomal, cardiac or central nervous system (CNS) abnormalities, and CNS infections [1]. Details of this registry have been published previously [1]. Full consent and ethical approval was given for the use of these tissues for research. Clinical details including the presence or absence of perinatal asphyxia, length of labour, and postnatal age have been documented [14]. Perinatal asphyxia was defined on the basis of an Apgar score of less than or equal to 5.0 at five minutes, a cord or initial blood pH of less than 7.1 and/or grade 2/3 encephalopathy. Brains were fixed in 20% formalin processed and paraffin embedded. The interval to postmortem was not documented. The cases were selected solely on the basis of presence (*n* = 12) or absence (*n* = 15) of pontine and/or hippocampal neuronal eosinophilia or karyorrhexis following review of routinely stained sections (haematoxylin and eosin). An anti-Apo J (clusterin) antibody (Santa Cruz Antibodies Cat no, SC-6419, Santa Cruz, CA, USA) was used at a dilution of 1 : 1000 after pretreatment with 0.01 M citrate buffer in the microwave for 3 × 5 minutes and visualised with diaminobenzidine. A wider survey of Apo J in other areas in the brain was undertaken in 2 of the positive cases and negative cases. Positive controls included biopsies from a Burkitt's lymphoma and Alzheimer's disease conditions in which clusterin expression has been previously described [16, 17]. Apo J assessment was undertaken in the hippocampus (including dentate fascia, CA1-4 sectors, subiculum, and entorhinal cortex) and pons (basis pontis and tegmentum including facial motor, abducens, trigeminal motor, and sensory nuclei) using a semiquantitative grading method (0 for no staining: + for 0–10% positive staining neurones; ++ for 10–40% positive staining neurones; +++ for over 50% of positive staining neurones) in the area examined. The entire section was screened in each case for the presence and location of any other Apo J-positive cells. Statistical analysis was carried out to compare the PSN group and controls using exact logistic regression, Fisher's exact test, Mann-Whitney *U*, and prism statistical software.

## 3. Results

### 3.1. Clinical Data

**Figure 1:**
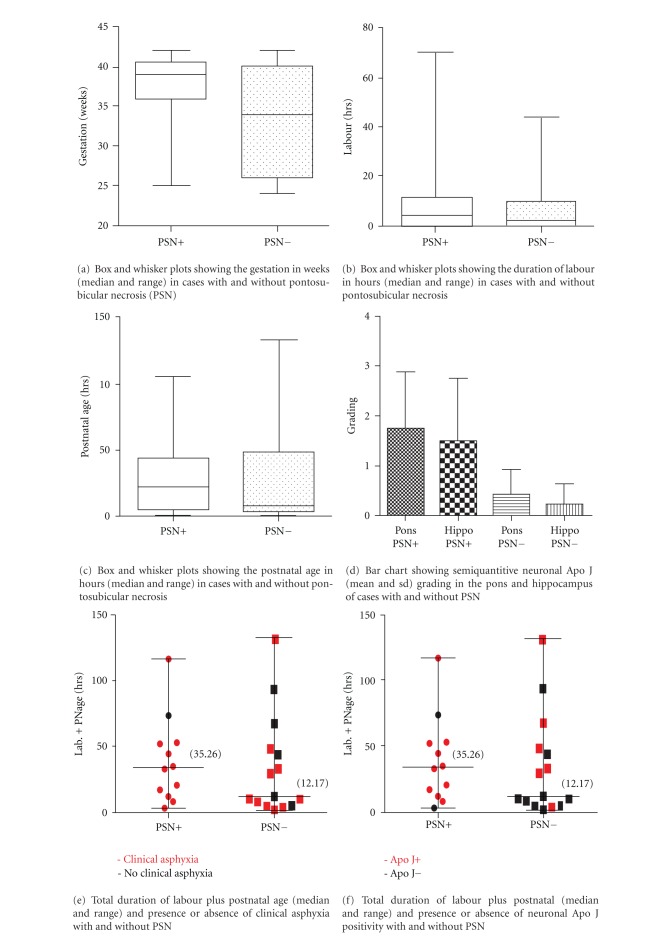
Box and whisker plots showing the gestation in weeks, duration of labour in hours, postnatal age in hours, semiquantitative neuronal Apo J, total duration of labour plus postnatal age in hours and presence or absence of asphyxia, and total duration of labour plus Postnatal Age and presence or absence of neuronal Apo J positivity with and without pontosubicular necrosis.

The median gestational age in the cases with PSN was 39 weeks (range 25–42 weeks) compared to 34 weeks (24–42) in controls without PSN, with no significant difference between groups (*P* = 0.584) (see Tables 1 and 2 and Figures 1(a), 1(b), and 1(c) for full clinical information). The median duration of labour was 4.44 hours (range 0–70.13 hours) in the PSN group compared with 1.62 hours (0–43.55) in controls, (*P* = 0.599); median postnatal age in the PSN group was 12.72 hours (0–51.13) compared with 7.98 hours (0.25–131.67) in controls, (*P* = 0.495); with no significant differences between groups. Clinical signs of asphyxia were present in 11/12 (91.7%) cases with PSN compared with 10/15 (66.7%) controls without PSN, with no significant difference between groups (*P* = 0.182).

### 3.2. Apo J Protein Expression

**Figure 2:**
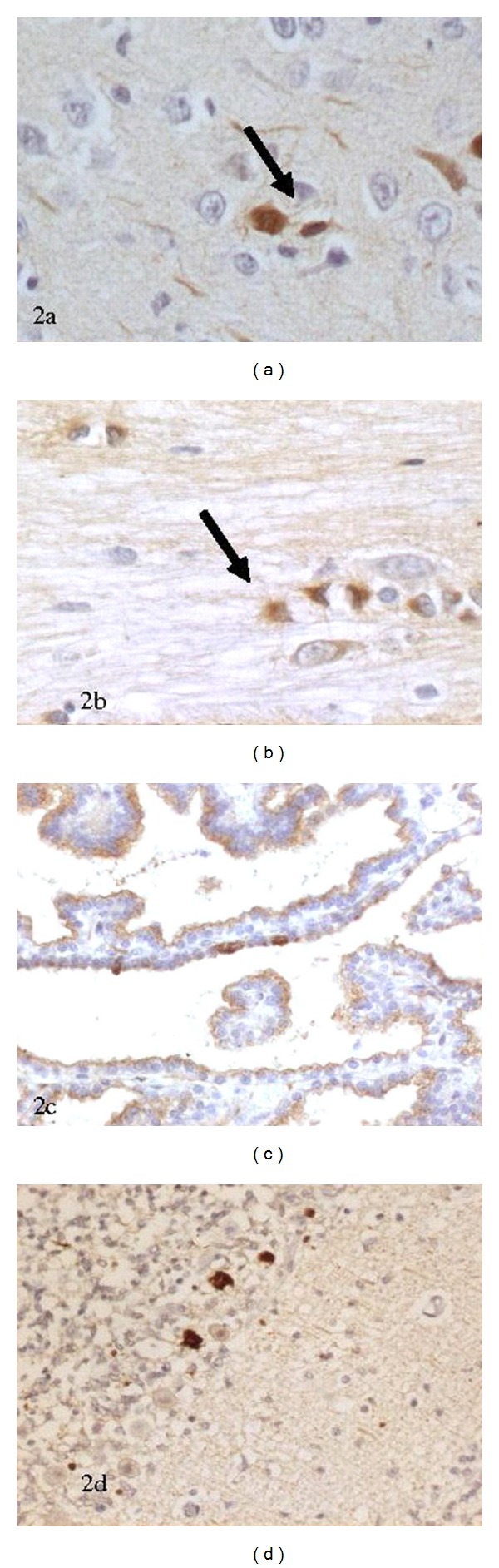
(a) Apo J neural positivity within viable and shrunken neurons. (b) Glial positivity within the base of the pons, respectively (×40) (c) Apo J neural positivity within the choroid plexus (×40) (d) Apo J positivity within the Purkinje cell layer (×40).

In the PSN cases 11/12 (92%) showed Apo J immunopositivity in neurons the base of the pons or hippocampus or both regions compared with 6/15 (40%) with Apo J neuronal positivity in the comparison group (*P* = 0.014, odds ratio 27.5, 95% confidence interval 2.881–262.48 using exact logistic regression)—independent of gestation, presence or absence of clinical asphyxia, duration of labour or postnatal age (see Table 2 and Figure 1(d) for semiquantitive grading). The positive neurones showed diffuse nuclear and cytoplasmic staining (Figure 2(a)). Apo J expression was present in neurones without histological evidence of damage as well as those with karyorrhexis and acidophilic cell change. In addition to Apo J expression in the pontine base neurons there were positive brainstem, neurones in the pontine tegmentum (facial motor, abducens, trigeminal motor, and sensory nuclei) in 9/12 (75%) in the PSN group compared with 5/15 (33%) in the comparison group (*P* = 0.054). 4/12 (33%) cases in the PSN group showed glial expression compared to 7/15 (46%) in the comparison group (*P* = 0.696). This positivity was localised to the astrocytic cell cytoplasm (Figure 2(b)). All the cases showed ependymal cytoplasmic positivity, which acted as a useful internal control. The choroid plexus, where present, showed cytoplasmic staining (Figure 2(c)). In two of the cases with pontosubicular necrosis and PSN negative cases, Apo J expression was examined in the basal ganglia, thalamus, frontal cortex, brainstem and cerebellum. Both cases with PSN showed Apo J neuronal expression in thalamic nuclei, cerebellar Purkinje cells (Figure 2(d)), brainstem nuclei, substantia nigra, and inferior olivary nuclei compared with the negative cases. Examination of Apo J positivity in clinically asphyxiated cases (Figures 1(e) and 1(f)) showed that 15/21 (71%) of clinically asphyxiated cases showed neuronal Apo J positivity compared with 2/6 (33%) of the cases with no evidence of clinical asphyxia (*P* = 0.154, odds ratio 5, 95% confidence interval 0.71 to 34.94).

## 4. Discussion

Our main finding is significant neuronal Apo J expression in 92% (11/12) of perinatal deaths with pontosubicular necrosis compared with 40% (6/15) of the cases without PSN (*P* = 0.014, odds ratio 27.5, 95% confidence interval 2.881–262.48)—independent of gestation, presence or absence of clinical asphyxia, duration of labour, or postnatal age.

In animal models of hypoxic/ischemic encephalopathy Apo J neuronal immunopositivity was detectable 12 hours after moderate hypoxic/ischaemic injury, becoming prominent at 18–24 hours after insult [18] with similar changes in mRNA in hippocampal lysates. In the clinical situation it is not possible to accurately time the onset of hypoxic/ischaemic damage to the brain and therefore the duration of labour and postnatal age are imperfect indicators of length of injury in our study. In addition, the time to postmortem was not documented in our cases, which may also affect immunopositivity. However it is interesting to note that in our PSN negative cases with Apo J positivity, the majority had a total duration of labour and postnatal age longer than 12 hours (Figure 1(f)), which is in keeping with previous animal data [18].

Our study also shows that a large proportion of clinically asphyxiated cases display no evidence of pontosubicular necrosis on histology (Figure 1(e)), in keeping with previous studies [1, 4]. Clinical signs of asphyxia did not correspond exactly with Apo J expression: 15/21 (71%) of clinically asphyxiated cases showed neuronal Apo J positivity compared with 2/6 (33%) of the cases with no evidence of clinical asphyxia (*P* = 0.154, odds ratio 5, 95% confidence interval 0.71 to 34.94) (Figures 1(e) and 1(f)). It is possible that clinical signs of asphyxia (i.e., Apgars, cord/blood pH, or encephalopathy) indicate hypoxic/ischaemia before protein or irreversible morphological changes have occurred within the brain. Previous studies have implicated clusterin in the response to hypoxic/ischaemic injury in the brain [10, 11], however it has also been described in dying neurons following status epilepticus [9] raising the possibility that is a marker of nonspecific marker of neuronal damage.

We also found Apo J positivity in the brainstem nuclei within the tegmentum of the pons 9/12 (75%) in the pontosubicular necrosis group and 5/15 (33%) in the control group (*P* = 0.054). Previous studies have shown that neurones within the trigeminal motor, facial, trigeminal somatosensory, and mesencephalic nuclei in particular contain high levels of Apo J mRNA [11].

Our study has shown Apo J glial positivity in the group with pontosubicular necrosis 4/12 (33%) and in the group without pontosubicular necrosis 7/15 (46%) with no significant difference between the groups (*P* = 0.696). Apo J mRNA has previously been demonstrated in astrocytes and neurones but not microglia [11]. Moreover in animal models of neonatal HI, there is initially an increase in Apo J mRNA and Apo J protein secreted by astrocytes, followed subsequently by Apo J protein accumulation in dying neurons [19, 20], raising the possibility that our glial Apo J immunopositivity represents an early response to ischaemic injury.

We found ependymal staining of Apo J in all cases and choroid plexus positivity when present (Figure 2(d)) acting as a useful positive internal control. Previous studies examining Apo J immunoreactivity in young adult rats, found that both protein and mRNA were high in the walls of the ventricles and over the choroid plexus [11, 12]. This staining may be a consequence either of protein synthesis or uptake from CSF. The apolipoprotein receptor, which has been shown to complex with Apo J for transport, is the gp 330/megalin receptor, which can be found on the ependymal lining of ventricles [21, 22].

Han et al. have suggested that in animal models Apo J contributes to caspase-3 independent brain injury or necrosis and have suggested that it may be a therapeutic target for modulating necrotic cell death following acute brain injury [14]. Interestingly, another study has found that nuclear neuronal Apo J increased in the cerebral cortex following acute ethanol treatment in developing rats and have implicated clusterin in the response to this mechanism of neuronal injury [15].

Our results raise the possibility that Apo J is similarly upregulated by hypoxia/ischaemia in the developing human brain, in keeping with animal models and previous studies [10, 11, 14, 15]. Larger follow-up studies are required to validate the findings.

## Figures and Tables

**Table 1:** Clinical information relating to early liveborn neonatal deaths with pontosubicular necrosis versus control group without pontosubicular necrosis.

Number of cases	PSN+	PSN−	Total
	12	15	27
Gestational age (weeks)			
Median	39	34	36
Range	(25–42)	(24–42)	(24–42)
Duration of labour (hours)			
Median	4.44	1.62	1.92
Range	(0–70.13)	(0–43.55)	(0–70.13)
Postnatal age (hours)			
Median	12.72	7.98	11.33
Range	(0–51.13)	(0.25–131.67)	(0.25–131.67)
Duration of labour plus postnatal age (hours)			
Median	23.76	12.17	29.33
Range	(2.25–115.67)	(2.27–131.67)	(2.25–131.67)
Presence of clinical asphyxia signs	11/12 (92%)	10/15 (67%)	21/27 (78%)

**Table 2:** Gestation, birth asphyxia, duration of labour, postnatal age, total duration of labour plus postnatal age, histology, and neuronal Apo J expression in the pontosubicular necrosis group and the group lacking pontosubicular necrosis.

PSN+ cases	Gestn weeks	Asphyxia (1 or 0)	Duration lab. (hours)	Postnatal age (hours)	Total lab. plus postnatal age (hours)	Pons HE (kary, eosin)	Hippoc. HE (kary/eosin)	Pons Apo J	Hippoc. Apo J
1	25	1	0.5	51.13	51.63	+	++	+	+++
2	27	0	70.13	3.37	73.5	+	+	++	+
3	36	1	0	7.97	7.97	++	++	+++	++
4	36	1	7.27	45.57	52.84	++	++	+++	+++
5	37	1	0	35	35	+++	+	+++	+
6	38	1	0	17	17	+	++	+	0
7	40	1	2	0.25	2.25	+	+	0	0
8	40	1	17.37	26.7	44.07	++	++	+++	+++
9	40	1	6.88	13.93	20.81	+	+	+	0
10	41	1	11.5	104.17	115.67	+	+	++	+++
11	41	1	0	32.52	32.52	+	+	++	+
12	42	1	11.17	0.63	11.8	+	+	0	+

PSN− cases	Gestn weeks	Asphyxia (1 or 0)	Duration lab. (hours)	Postnatal age (hours)	Total labour plus postnatal age (hours)	Pons HE	Hippoc. HE	Pons Apo J	Hippoc. Apo J

1	24	1	0.58	7.98	8.56	0	0	0	0
2	24	1	0	4.42	4.42	0	0	+	0
3	25	1	1.62	3.13	4.75	0	0	0	0
4	26	1	18	11.33	29.33	0	0	+	+
5	28	0	0	66.87	66.87	0	0	+	+
6	27	1	0	131.67	131.67	0	0	+	0
7	32	0	8.42	3.75	12.17	0	0	0	0
8	34	0	6.83	86.25	93.08	0	0	0	0
9	35	1	0	10.68	10.68	0	0	0	0
10	36	0	43.55	0.28	43.83	0	0	0	0
11	36	1	0	48	48	0	0	+	+
12	40	1	9.95	0.25	10.2	0	0	0	0
13	40	1	1.92	0.35	2.27	0	0	0	0
14	40	1	10.48	22.68	33.16	0	0	+	0
15	42	0	0	4.88	4.88	0	0	0	0
